# Brucellosis involving the aorta and iliac arteries: a systematic review of 130 cases

**DOI:** 10.3389/fbioe.2023.1326246

**Published:** 2023-11-30

**Authors:** Xiao Li, Xiaoyu Li, Zhihua Cheng

**Affiliations:** ^1^ Department of Vascular Surgery, General Surgery Center, The First Hospital of Jilin University, Changchun, China; ^2^ Department of Otolaryngology, The First Hospital of Jilin University, Changchun, Jilin, China

**Keywords:** brucellosis, review, aorta and iliac arteries, endovascular therapy, infection

## Abstract

**Objective:** Brucellosis, the most common bacterial zoonosis, poses a serious threat to public health in endemic regions. Cardiovascular complications of brucellosis, mostly pericarditis or endocarditis, are the leading cause of brucellosis-related death. Complications involving the aorta and iliac arteries are extremely rare but can be life-threatening. Our objective was to identify and review all reported cases of aortic and iliac involvement in brucellosis to provide a deep, up-to-date understanding of the clinical characteristics and management of the disease.

**Methods:** Online searches in PubMed, Web of Science, China National Knowledge Infrastructure, and the Chinese Wanfang database were conducted to collect articles reporting cases of brucellosis with aortic and iliac artery involvement. All data in terms of patient demographics, diagnostic methods, clinical manifestations, and treatment regimens and outcomes were extracted and analyzed in this systematic review.

**Results:** A total of 79 articles were identified, reporting a total of 130 cases of brucellosis with aortic and iliac artery involvement. Of the 130 cases, 110 (84.5%) were male individuals and 100 (76.9%) were over 50 years old. The patients had an overall mortality rate of 12.3%. The abdominal aorta was most commonly involved, followed by the ascending aorta, iliac artery, and descending thoracic aorta. Arteriosclerosis, hypertension, and smoking were the most common comorbidities. There were 71 patients (54.6%) who presented with systemic symptoms of infection at the time of admission. Endovascular therapy was performed in 56 patients (43.1%), with an overall mortality rate of 3.6%. Open surgery was performed in 52 patients (40.0%), with an overall mortality rate of 15.4%.

**Conclusion:** Aortic and iliac involvement in brucellosis is extremely rare but can be life-threatening. Its occurrence appears to be associated with the male gender, an older age, arteriosclerosis, and smoking. Although the number of reported cases in developing countries has increased significantly in recent years, its incidence in these countries may still be underestimated. Early diagnosis and therapeutic intervention are critical in improving patient outcomes. Endovascular therapy has become a preferred surgical treatment in recent years, and yet, its long-term complications remain to be assessed.

## Introduction

Brucellosis is a zoonotic infection caused by the bacterial genus *Brucella*. It is the most common bacterial zoonosis, with more than 500,000 cases reported worldwide each year (Herrick et al., 2014; Franco et al., 2007). Most human and animal cases of brucellosis occur in resource-poor developing countries, especially those in the Middle East, Mediterranean, and East Asia. Brucellosis is mainly contracted through occupational exposure of laboratory or slaughterhouse workers to infected animals, consumption of infected, unpasteurized dairy products or contaminated meat products, or inhalation of Brucella-containing aerosols (Pappas et al., 2005). The general systemic symptoms include fever, fatigue, and joint and muscle pain. Brucellosis can also cause organ-specific symptoms involving the liver, central nervous system, genitourinary system, or circulatory system (Pappas et al., 2005). The damage to the circulatory system, most often presenting as pericarditis or endocarditis, occurs in 3% of cases. Although the overall fatality rate of brucellosis is approximately 1%, and endocarditis occurs in only 1%–2% of cases, 80% of brucellosis-related deaths are associated with endocarditis (Buzgan et al., 2010; Dean et al., 2012; Li et al., 2021; Jin et al., 2023). An infected aneurysm is a life-threatening condition caused by an abnormal swelling or bulge in the wall of an artery due to an infection that destroys the inner lining of the artery. The bacteria that most commonly cause these infections are *Salmonella*, *Staphylococcus*, and *Streptococcus*. Infected aneurysms involving the aorta and iliac arteries, although extremely rare, can occur in the course of brucellosis (Herrick et al., 2014; Willems et al., 2022). If left untreated, aneurysms can rupture quickly, causing severe bleeding and even death (Müller et al., 2001; Luo et al., 2003; Brossier et al., 2010; Jiang et al., 2023). In 2022, Willems and colleagues published a review on aortic and iliac involvement in brucellosis, wherein they conducted PubMed, Web of Science, and AccessMedicine searches and identified (Ramachandran Nair et al., 2019) cases with an overall mortality rate of 22% (Willems et al., 2022).

Thanks to the increasingly common practice of brucellosis diagnosis and treatment in China, the number of reports on brucellosis involving aorta and iliac arteries in Chinese patients has increased significantly in recent years (Wang et al., 2021; Yu and Zhao, 2021; Ma et al., 2023). To expand the discoveries of Willems et al. (2022), we conducted online searches in the China National Knowledge Infrastructure (CNKI) and the Chinese Wanfang database. As a result, we identified a total of (Liu and ZY, 2022) 79 articles reporting a total of 130 cases of brucellosis with aortic and iliac artery involvement. Our objective was to identify and review all reported cases of aortic and iliac involvement in brucellosis to provide a deep, up-to-date understanding of the clinical characteristics and management of the disease.

## Materials and methods

### Literature review

**FIGURE 1:**
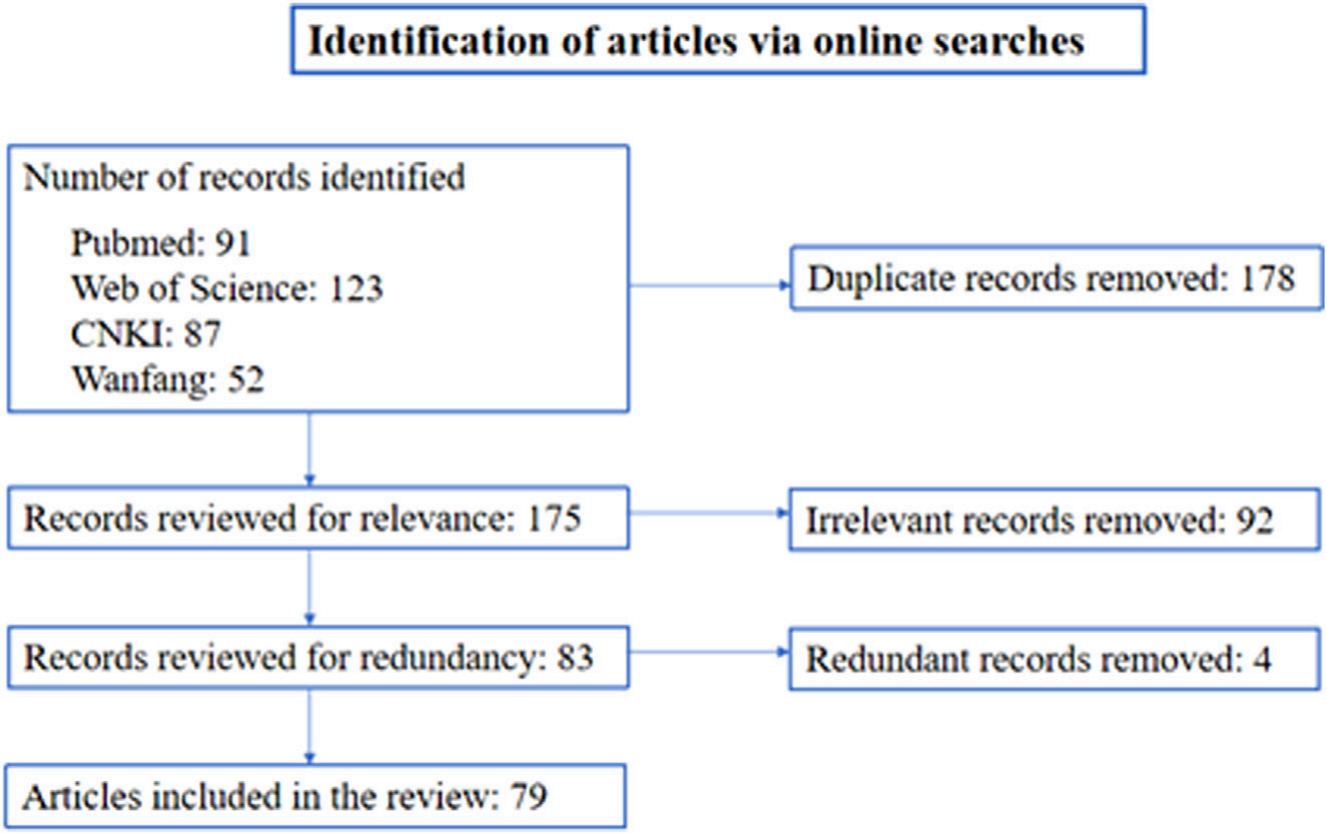
Flow diagram of the search strategy for articles on aortic and iliac involvement in brucellosis.

To identify reports on aortic and iliac involvement in brucellosis, we conducted searches in PubMed, Web of Science, CNKI, and the Chinese Wanfang database using terms (*Brucella* or brucellosis) AND (abdominal/thoracic aneurysm or pseudoaneurysm or aortitis or aorta or mycotic). The searches were performed without restriction on language and with any date of publication before 1 August 2023. The retrieved articles were included for meta-analysis if they reported on aortic and iliac artery involvement in brucellosis. We also hand-searched references contained in the retrieved articles. We included all articles that provided information on the aorta or iliac artery pathology caused by brucellosis, including cohort studies, case–control studies, case reports, and case series. Aorta and iliac artery pathology included infected aneurysms (pseudoaneurysms and true aneurysms), arterial dissection, abscesses, interwall hematoma, and ulcers. A quality assessment was not used to exclude articles. Cases involving peripheral or cerebral arteries, isolated endocarditis, and duplicate cases were excluded. All articles identified were independently reviewed for relevance by two authors (Xiao Li and Zhihua Cheng). A flow chart showing search strategies and results is presented in Figure 1.

### Clinical data

If available, the following information was extracted from each article: reference name, the year and country of publication, gender, age, route of transmission, country of transmission, comorbidities, clinical symptoms and course of the disease, *Brucella* species, diagnostic tests (blood cultures, biopsy cultures, or serological tests) and results, the location and type of aortic and iliac artery manifestations, manifestations at other sites (especially endocarditis, intervertebral discitis, psoas abscess, and fistula), antibiotic treatment (dose and duration), surgical treatment, and end results (follow-up and complications). Since not every article provided information on every variable, the denominator of a single variable may be less than the total number of cases enclosed for the meta-analysis.

### Statistical methods

SPSS 26.0 software was used for statistical analysis. Continuous variables are presented as mean ± standard deviation (SD). Count data are presented as number and percentage [n (%)].

## Results

### Articles and cases

A total of 79 articles reporting on 130 cases of human brucellosis involving the aorta and iliac arteries were identified and included in the meta-analysis (Herrick et al., 2014; Willems et al., 2022; Jiang et al., 2023; Yu and Zhao, 2021; Wang et al., 2023; Cascio et al., 2012; Hart et al., 1951; Peery and Belter, 1960; Bennett, 1967; Golden et al., 1970; Quilichini et al., 1974; Cros and Maestracci, 1975; Fudge et al., 1977; Colonna and Cristallo, 1978; Gillet et al., 1983; Cueto García et al., 1983; Aguado et al., 1987; Pressl et al., 1990; Kasab et al., 1990; Bergeron et al., 1992; Kumar et al., 1993; Gross et al., 1994; Sanchez-Gonzalez et al., 1996; Yee and Roach, 1996; Blain et al., 1997; Cano Trigueros et al., 1997; Peláez Fernández and Sánchez Galindo, 1999; Shehata and Adib, 2001; Rousié et al., 2004; Alhyari et al., 2005; Quaniers et al., 2005; Tsioufis et al., 2006; Biyik et al., 2007; Bul et al., 2007; Park et al., 2007; Kusztal et al., 2007; Kokkinis et al., 2008; Wolff et al., 2009; Ahmed et al., 2010; Bakhos et al., 2010; Jun Park and Hyun Cho, 2010; Kwon et al., 2010; Sasmazel et al., 2010; Amirghofran et al., 2011; Benedetto et al., 2011; Jariwala, 2013; Goudard et al., 2013). There were 65 single-case reports, 6 articles reporting on 2 cases, 1 article reporting on 3 cases, 2 articles reporting on 4 cases, 1 article reporting on 5 cases, 1 article reporting on 9 cases, 1 article reporting on 13 cases, and 1 article reporting on 15 cases. The combined cases involved all aortic segments. The clinical features, diagnostic tests, treatments, and outcomes of each case are shown in Supplementary Table S1.

### Gender distribution

Of the 130 cases of brucellosis involving the aorta and iliac arteries, one case did not provide information on the gender of the patient. Of the 129 cases that did specify the gender of the patient, 109 (84.5%) were male and 20 (15.5%) were female individuals (Table 1).

**TABLE 1:** Brucellosis involving the aorta and iliac arteries: patient demographics, comorbidity, epidemiological history, and diagnostic method.

	Cases (*n* = 130)
Age (years (range))	58.3 (11.0–83.0)
Age distribution (years)	
40 or younger	16 (12.4%)
41–50	14 (10.9%)
51–60	23 (17.8%)
61–70	54 (41.9%)
71–80	20 (15.5%)
Over 80	2 (1.6%)
Gender	
Male	109 (84.5%)
Female	20 (15.5%)
Regional distribution	
China	60 (50.4%)
Mediterranean countries	29 (24.4%)
The United States	11 (9.2%)
Saudi Arabia	8 (6.7%)
Northern Europe	7 (5.9%)
Iran	4 (3.4%)
Comorbidity	
Arteriosclerosis	27 (20.8%)
Smoking history	24 (18.5%)
Hypertension	21 (16.2%)
Alcoholism	10 (7.7%)
Diabetes	3 (2.3%)
Coronary atherosclerotic heart disease	1 (0.8%)
Cerebral infarction	2 (1.5%)
Hyperlipidemia	3 (2.3%)
Epidemiological history	
Animal contact	50 (38.5%)
Unpasteurized milk	16 (12.3%)
Occupational exposure	50 (38.5%)
Diagnostic method	
Serological test	110 (84.6%)
Positive	104 (94.5%)
Negative	6 (5.5%)
Blood culture test	108 (83.1%)
Positive	64 (59.3%)
Negative	44 (40.7%)
Biopsy culture test	46 (35.4%)
Positive	33 (71.7%)
Negative	13 (28.3%)

Data are presented as n (%). Each case may have multiple comorbidities and diagnostic methods.

### Age distribution

Age was not specified in one of the 130 cases. The patient age in the other 129 cases ranged from 11 to 83 years, with a mean age of 58.3. There were 16 patients who were 40 years old or younger (12.4%), 14 patients who were 41–50 years old (10.9%), 23 patients who were 51–60 years old (17.8%), 54 patients who were 61–70 years old (41.9%), 20 patients who were 71–80 years old (15.5%), and two patients who were over 80 years old (1.6%) (Table 1). The age group of 61–70 years had the highest incidence of common iliac artery involvement.

### Regional distribution

China had the greatest number of patients [(Korkut et al., 2015), 50.4%], followed by Mediterranean countries (29, 24.4%), the United States (11, 9.2%), Saudi Arabia (8, 6.7%), Northern Europe (7, 5.9%), and Iran (4, 3.4%) (Table 1). The regions of residence of the remaining 11 cases were not reported.

### Comorbidity

The most common comorbidities were arteriosclerosis (27, 20.8%), smoking history (24, 18.5%), and hypertension (21, 16.2%) (Table 1). Other comorbidities included alcoholism (10, 7.7%), diabetes (3, 2.3%), coronary atherosclerotic heart disease (1, 0.8%), cerebral infarction (2, 1.5%), and hyperlipidemia (3, 2.3%).

### Epidemiological history

A summary of the patient epidemiological history is presented in Table 1. Out of the 130 patients, 50 (38.5%) had a history of animal contact, including 26 with sheep, 13 with cattle, 5 with cattle and sheep, 3 with wild boar, 2 with cats and dogs, and 1 with camels. Sixteen patients (12.3%) consumed unpasteurized milk products. Fifty patients (38.5%) had a risk of occupational exposure to infected animals, including 20 farmers, 7 animal keepers, 6 travelers, 5 factory workers, 4 butchers, 3 veterinarians, 2 wild boar hunters, 2 cooks, and 1 herdsman. Eleven patients contracted brucellosis abroad but were diagnosed and treated in their country of residence.

### Overview of clinical presentation

A total of 71 patients (54.6%) showed one or more general systemic symptoms of infection at the time of admission. The common symptoms were fever (61, 46.9%), weight loss (17, 13.1%), and fatigue (13, 10.0%). Less common symptoms included hypothermia, nausea, vomiting, and palpitation. More specific symptoms depend on the site of blood vessel involvement. Involvement of the abdominal aorta was mainly manifested as abdominal pain, lower back pain, and pulsating abdominal mass. Hypovolemic shock occurred in certain cases of abdominal aorta rupture. Involvement of the thoracic aorta presented with chest pain and back pain. Additional aorta manifestations included aortoesophageal fistula with hematemesis, aortotracheal fistula with hemoptysis, and aortointestinal fistula with hematochezia. Involvement of the iliac artery mainly presented with groin pain and lower abdominal pain.

### Diagnostic tests

Human *Brucella* infections can be diagnosed by serological tests, blood culture, or biopsy culture. Serological tests include the Coombs test, serum agglutination test, and enzyme-linked immunosorbent assay (ELISA). In the current review, serological tests were conducted in 110 patients (84.6%), making them the most commonly used diagnostic method in the meta-analysis. Of these 110 patients, 104 (94.5%) tested positive and 6 (5.5%) tested negative. A total of 108 patients (83.1%) undertook the blood culture test, of whom 64 (59.3%) were positive and 44 (40.7%) were negative. Additionally, a total of 46 patients (35.4%) undertook the biopsy culture test, of whom 33 (71.7%) were positive and 13 (28.3%) were negative. A summary of diagnostic methods and results is presented in Table 1. Nineteen patients (14.6%) were diagnosed to have brucellosis before admission, and for the other 111 patients, the diagnosis was made after admission.

### *Brucella* species

*Brucella* species were reported in 44 cases (33.8%), of which *Brucella melitensis* (*B. melitensis*) was the most common (32, 72.7%), followed by *B. abortus* (7, 15.9%) and *B. suis* (5, 11.4%).

### Vessel involvement sites and pathological manifestations

The abdominal aorta was the most common site of involvement (82 cases, 63.1%), followed by the ascending aorta (28, 21.5%), iliac artery (22, 16.9%), and descending thoracic aorta (14, 10.8%) (Table 2). There were 67 cases (51.5%) that involved the abdominal aorta alone, 28 cases (21.5%) that involved the ascending aorta alone, 10 cases (7.7%) that involved the descending thoracic aorta alone, and 10 cases (7.7%) that involved the iliac artery alone, with three in the right, six in the left, and one in the bilateral iliac artery. There were 14 patients (10.8%) who had two sites of involvement, with three in the descending thoracic aorta and abdominal aorta, six in the abdominal aorta and right iliac artery, and five in the abdominal aorta and bilateral iliac artery. One patient (0.8%) had three sites of involvement, which were the descending thoracic aorta, abdominal aorta, and iliac artery.

**TABLE 2:** Brucellosis involving the aorta and iliac arteries: vessel involvement sites.

Involvement site	Cases (*n* = 130)
Aorta	
Ascending aorta	28 (21.5%)
Abdominal aorta	82 (63.1%)
Descending thoracic aorta	14 (10.8%)
Iliac artery	22 (16.9%)

Data are presented as n (%). Each case may have multiple involvement sites.

In terms of pathological manifestations of the blood vessel, infected pseudoaneurysm was the most commonly diagnosed (69, 53.1%), followed by infected true aneurysm (41, 31.5%), ascending aortic root abscess (8, 6.2%), arteritis (7, 5.4%), dissecting aneurysm (5, 3.8%), aortic ulcer (5, 3.8%), interwall hematoma (2, 1.5%), and thrombotic occlusion of the aorta (1, 0.8%) (Table 3). In the cases that involved the abdominal aorta, there were 49 cases of infected pseudoaneurysm, 28 cases of infected true aneurysm, 3 cases of aortitis, 1 case of dissecting aneurysm, 1 case each of aortic ulcer and interwall hematoma, and 1 case of thrombotic occlusion of the aorta. In the cases that involved the descending thoracic aorta, there were five cases each of infected pseudoaneurysm and true aneurysm and one case each of dissecting aneurysm, interwall hematoma, aortitis, and aortic ulcer. In the cases that involved the ascending aorta, there were eight cases of root abscess, five cases each of infected pseudoaneurysm and true aneurysm, four cases each of dissecting aneurysm and aortic ulcer, and three cases of arteritis. In the cases that involved the iliac artery, there were 12 cases of infected pseudoaneurysm, nine cases of infected true aneurysm, and one case of dissecting aneurysm.

**TABLE 3:** Brucellosis involving the aorta and iliac arteries: pathological manifestations of vessel involvement.

Pathological manifestation	Cases (*n* = 130)
Infected pseudoaneurysm	69 (53.1%)
Infected true aneurysm	41 (31.5%)
Ascending aortic root abscess	8 (6.2%)
Arteritis	7 (5.4%)
Dissecting aneurysm	5 (3.8%)
Aortic ulcer	5 (3.8%)
Interwall hematoma	2 (1.5%)
Thrombotic occlusion of the aorta	1 (0.8%)

Data are presented as n (%). Each case may have multiple pathological manifestations.

### Complications

A summary of complications is presented in Table 4. Endocarditis (24, 18.5%), discitis (13, 10.0%), fistula formation (12, 9.2%), and psoas abscess (6, 4.6%) were the most common complications. The complications that occurred at a lower incidence rate included splenic infarction (5, 3.8%), thrombosis (4, 3.1%), inguinal lymph node enlargement (3, 2.3%), osteomyelitis (1, 0.8%), and epididymitis (1, 0.8%). All endocarditis occurred in cases involving the ascending aorta except one, which occurred in a case involving the abdominal aorta. Of the 13 cases of discitis, 12 occurred in cases involving the abdominal aorta or iliac artery and 1 occurred in a case involving the descending thoracic aorta. All psoas abscesses and abdominal lymph node enlargement complications occurred in cases involving the abdominal aorta, and all splenic infarction occurred in cases involving the ascending aorta. Fistula formation occurred in six cases involving the ascending aorta, with two between the aortic sinuses, two on the right ventricle, one between the aorta and pulmonary artery, and one on the aortic wall. Fistula formation also occurred in five cases involving the descending thoracic aorta, with three aortotracheal and two aortoesophageal fistulas. Finally, an aortoduodenal fistula was reported in a case involving the abdominal aorta.

**TABLE 4:** Brucellosis involving the aorta and iliac arteries: complications.

Complication	Cases (*n* = 130)
Endocarditis	24 (18.5%)
Discitis	13 (10.0%)
Fistula formation	12 (9.2%)
Psoas abscess	6 (4.6%)
Splenic infarction	5 (3.8%)
Thrombosis	4 (3.1%)
Remaining	5 (3.8%)

Data are presented as n (%). Each case may have multiple complications.

### Antibiotic treatment

Overall, the antibiotic treatment was a combination of fluoroquinolones, sulfonamides, tetracycline, rifampicin, and aminoglycosides. The specific treatment combination, dose, and duration varied from one medical center to another. In articles published over the past 10 years, intravenous gentamicin in combination with oral doxycycline and rifampicin was the most commonly used treatment regimen. The duration of treatment varied from 6 weeks to a lifetime.

### Surgical treatment

**FIGURE 2:**
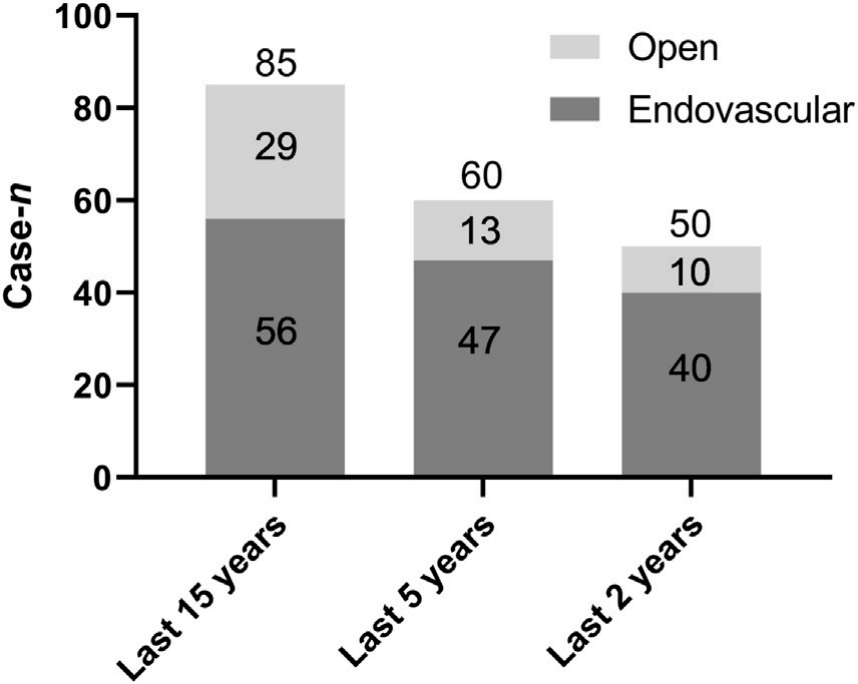
Brucellosis involving the aorta and iliac arteries: surgical treatments (open vs. endovascular) within the past 15, 5, and 2 years.

Of the 130 cases, 56 (43.1%) received endovascular surgery only, 52 (40.0%) received open surgery only, 4 (3.1%) received open surgery after endovascular surgery, and 15 (11.5%) adopted conservative treatment without surgery. Information on surgery was not provided in the remaining three cases (2.3%). In 2007, endovascular surgeries were first reported to treat infection of the iliac artery caused by brucellosis. Of the 99 cases reported since 2007 (within the past 15 years), 56 (56.6%) received endovascular surgery and 29 (29.3%) received open surgery. Of the 68 cases reported over the past 5 years, 47 (69.1%) received endovascular surgery, 13 (19.1%) received open surgery, and 3 (4.4%) received open surgery after endovascular therapy. Of the 54 cases reported over the past 2 years, 40 (74.1%) received endovascular surgery and 10 (18.5%) received open surgery. Endovascular surgery has become a preferred surgical treatment option over the years (Figure 2).

Of the 28 cases involving the ascending aorta, 17 (60.7%) received surgical treatment (all open surgical procedures, including three Bentall surgery cases), 9 (33.3%) went without surgery, and the information on surgery was not provided for the two remaining cases. Arterial ulcers were mainly treated with valve replacement, and abscesses were treated with antibiotics and/or abscess drainage. Of the 11 cases of infected aneurysms of the ascending aorta, eight were treated with aneurysm resection and valve replacement, one received Bentall surgery, and the information on surgery was not provided for the remaining two cases.

Of the 14 cases involving the descending thoracic aorta, 13 received surgical treatment, including eight endovascular surgery procedures, three open surgery procedures, and two open surgery procedures after endovascular surgery. The information on surgery was not provided for the remaining case. Of the 12 cases of infected aneurysm of the descending thoracic aorta, seven received thoracic endovascular aortic repair surgery (TEVAR), three underwent aneurysm resection and artificial vascular replacement, and two received open surgery for fistula complications after endovascular therapy. TEVAR was performed in one case of aortic dissection. Of the 82 cases involving the abdominal aorta, 74 were treated with surgery (40 intracavitary surgery procedures, 33 open surgery procedures, and 1 open surgery after endovascular surgery), 6 did not undergo surgery, and the information on surgery was not provided for the remaining two cases. Of the 33 patients who underwent open surgery for infected aortic aneurysm, 22 received abdominal aortic aneurysm resection and artificial vascular replacement and 5 underwent aneurysm resection and axillary-bilateral femoral artery bypass. Endovascular therapy was performed in 38 cases of infected abdominal aortic aneurysm.

Of the 10 cases of iliac artery involvement alone, nine were treated with endovascular isolation technology. The remaining case received drainage treatment for abscesses surrounding the stent and autologous vein reconstruction of the aorta and iliac arteries after endovascular treatment.

### Outcome and follow-up

Of the 130 cases, 91 (70.0%) reported a specific follow-up time ranging from 9 days to 8 years, with an average time of 16.6 months. During the follow-up, 106 (81.5%) patients were considered cured by antibiotic and/or surgical treatment, 16 (12.3%) died after treatment, 7 (5.4%) had no significant follow-up records, and 1 (0.8%) was lost to follow-up. The main causes of death were bleeding, multi-organ failure, and shock. Of the 112 patients who underwent surgery, 10 (8.9%) died after surgery, including two out of 56 who received endovascular surgery and eight out of 52 who received open surgery. Notably, four patients underwent open surgery after endovascular surgery. The overall rate of secondary surgery was 5.4% (6/112). Of the 15 patients who received conservative treatment, six (40.0%) died.

## Discussion

*Brucella* bacteria can replicate in a variety of mammalian cell types, including microglia, fibroblasts, epithelial cells, and endothelial cells (Wang et al., 2023; Martirosyan and Gorvel, 2013), causing multiple organ damage. Brucellosis involving the aorta and iliac arteries is extremely rare but can be life-threatening (Herrick et al., 2014; Willems et al., 2022; Cascio et al., 2012). In 2022, Siem and colleagues published a review on this severe complication of brucellosis (Jin et al., 2023), wherein a total of 71 cases were identified through searches in PubMed, Web of Science, and AccessMedicine. The 71 cases had an overall mortality rate of 22%, and the highest number of cases reported in a single article was three. The past 2 years have witnessed an increased clinical awareness of brucellosis and its complications in developing countries, such as China, resulting in a greater number of reports from these countries on this disease. In this review, we identified 130 cases of brucellosis involving aorta and/or iliac arteries through searches in PubMed, Web of Science, CNKI, and the Chinese Wanfang database. The 130 cases were gathered from 79 relevant articles, including a 14-patient 16 and a 15-patient 12 single-center clinical case study. Notably, both centers were located in China. This suggested that the incidence of this life-threatening complication of brucellosis likely has been underestimated in developing countries due to insufficient diagnosis, as well as scientific and financial barriers to publication.

In the current review, 76.8% of patients were over 50 years old and 84.5% were men. This is consistent with that degenerative aneurysm is associated with advanced age and the male gender (Hannawa et al., 2009). Arteriosclerosis (20.8%), hypertension (16.2%), and smoking history (18.5%) were the most common comorbidities. This suggests that blood vessel degeneration due to arteriosclerosis, hypertension, or smoking makes the aorta and iliac arteries more vulnerable to *Brucella* infection. Infection of the common iliac artery can be caused by bacterial spread from the site of entry or pathogen introduction as a result of improper operation that causes damage to the artery wall. Previous studies have found that the presence of atherosclerotic plaques in the abdominal aorta, carotid arteries, or femoral arteries doubles the risk of infected aneurysms (Wang et al., 2023). A critical pathological hallmark of aneurysm in this patient group is the necrosis and/or rupture of atherosclerotic blood vessel walls, leading to bacterial adhesion and spread. Atherosclerotic plaques have irregular surfaces that provide potential attachment sites for microbes (Betancourt et al., 2007).

In the current review, endocarditis [(Fudge et al., 1977), 18.5%], discitis [(Yu and Zhao, 2021), 10.0%], psoas abscess [(Li et al., 2021), 4.6%], and fistula formation [(Jiang et al., 2023), 9.2%] were the most common complications. Nearly all endocarditis complications (23 out of 24) occurred in cases involving the ascending aorta, and only one occurred in a case involving the abdominal aorta. Of the 13 cases of discitis (Jiang et al., 2023), 12 occurred in patients with abdominal and iliac artery involvement and only one occurred in a patient with descending thoracic aorta involvement. All psoas abscesses and abdominal lymph node enlargement complications occurred in cases involving the abdominal aorta. These complications likely resulted from direct bacterial spread from the abdominal aorta to the anterior surface of the cone and psoas muscle, which are structurally adjacent to the aorta. Fistula formation occurred in six cases involving the ascending aorta, with two between the aortic sinuses, two on the right ventricle, one between the aorta and pulmonary artery, and one on the aortic wall. Fistula formation also occurred in five cases involving the descending thoracic aorta, with three aortotracheal and two aortoesophageal fistulas. Additionally, an aortoduodenal fistula was reported in a case involving the abdominal aorta. Fistula formation is a result of chronic damage to blood vessels caused by *Brucella* bacteria localized on the surface of the vessel wall. Fistulas, which can be considered tubes connecting adjacent arteries, can cause bleeding or even shock, which may result in death. In clinical practice, the presence of hemoptysis or gastrointestinal bleeding signals the possibility of fistula complications and calls for timely diagnosis and treatment.

Similar to previous reports (Herrick et al., 2014; Willems et al., 2022; Cascio et al., 2012), the main systemic symptoms were latent fever, weight loss, fatigue, and joint pain. Notably, only 54.6% of patients (71/130) showed systemic symptoms of infection at the time of admission, which may be a result of the use of over-the-counter antipyretic analgesics. The difficult-to-track epidemiological contact history of patients and the lack of specific systemic symptoms are considerable challenges to the early diagnosis of brucellosis. In the current review, only 14.6% of patients (19/130) were diagnosed before admission. The vast majority of patients already presented with clinical manifestations of aortic and iliac artery involvement at the time of admission before brucellosis was diagnosed. The most common site of involvement was the abdominal aorta (82/130, 63.1%), followed by the ascending aorta (28/130, 21.5%), iliac artery (22/130, 16.9%), and descending thoracic aorta (14/130, 10.8%). The specific clinical symptoms varied depending on the site of involvement. Involvement of the abdominal aorta was mainly manifested as abdominal pain, lower back pain, and pulsating abdominal mass. Hypovolemic shock occurred in certain cases of abdominal aorta rupture. Involvement of the thoracic aorta presented with chest pain and back pain. Additional aorta manifestations included aortoesophageal fistula with hematemesis, aortotracheal fistula with hemoptysis, and aortointestinal fistula with hematochezia. Involvement of the iliac artery mainly presented with groin pain and lower abdominal pain. The duration of symptoms ranged from a few days to years.

*Brucella* infection can be diagnosed by serological tests, blood culture, or biopsy culture. Serological tests are generally more sensitive than the culture methods, and hence, they are most commonly used in the diagnosis of brucellosis (Jiang et al., 2023). For example, 92% of patients with *Brucella* arteritis tested positive in either the agglutination or ELISA serological test 1. In the current review, 84.6% (110/130) of patients undertook a serological test, of whom 94.5% (104/110) tested positive. Culture tests are less sensitive and require weeks of culture time (Willems et al., 2022; Araj et al., 1986). Blood cultures in most hospitals are usually discarded after 5–7 days, which is not long enough to detect the slow-growing *Brucella* bacteria. Previous reviews found that the average sensitivity of the blood culture test for brucellosis was only 68% 1. In the current review, 83.1% of patients (108/130) undertook the blood culture test, of whom 64 tested positive, giving an average sensitivity of 59.3%. The biopsy culture test is mainly used for patients who undergo open surgery. Over the recent years, the utilization of this culture method has gradually waned as endovascular therapy has become the preferred surgical treatment. In the current review, 35.4% of patients (46/130) undertook the biopsy culture test, of whom 33 tested positive, giving an average sensitivity of 71.7%. Early diagnosis of brucellosis may help raise awareness of possible severe complications, such as *Brucella* aneurysm, and hence encourage early intervention and save lives. Yet, the lack of access to appropriate diagnostic tests for brucellosis in hospitals in developing countries almost certainly leads to underdiagnosis of the infection. To increase the rate of diagnosis, hospitals need to extend the culture time to allow adequate bacterial expansion. In addition to serological or culture tests, epidemiological contact history and clinical manifestations need to be taken into account for treatment decision-making.

Combinatorial antibiotic therapy is the gold standard for treating brucellosis. Monotherapy and treatment durations of less than 6 weeks are associated with high rates of recurrence and complications and, hence, are not acceptable treatment strategies (Herrick et al., 2014; Willems et al., 2022). The Sanford Guide to Antimicrobial Therapy recommends 100 mg of doxycycline twice daily for 6 weeks and 5 mg/kg of gentamicin once daily for 1 week for brucellosis in the absence of local lesions (Fever Sanford Guidelines, 2021). The World Health Organization recommends 100 mg of doxycycline twice daily and 600–900 mg of rifampicin once daily for 6 weeks as standard treatment, and doxycycline for 6 weeks and streptomycin for the first 3 weeks as an acceptable alternative (Joint FAO, 1986). According to Harrison’s Infectious Diseases, the gold standard treatment for adults is intramuscular 0.75–1 g of streptomycin once daily for 14–21 days in combination with 100 mg of doxycycline twice daily for 6 weeks (Kasper et al., 2019). According to the Expert Consensus on the Diagnosis and Treatment of Brucellosis formulated in China in 2017, antibiotic treatment should continue for at least 3–6 months in the presence of comorbidity (Diseases EBoCJoI, 2017). In the current review, although the antibiotic combination and dose varied depending on the location of the admission hospital, patients were typically treated with a combination of two or three of five classes of antibiotics, namely, fluoroquinolones, sulfonamides, tetracycline, rifampin, and aminoglycosides. The treatment duration varied from 6 weeks to a lifetime, with 3 months being the most common.

Surgical treatment of brucellosis involving the aorta and iliac arteries, which includes open surgery and endovascular therapy, is aimed at complete clearance of the infected area, reconstruction of the blood vessels, and restoration of the blood flow. Open surgery has long been recognized as the gold standard in the treatment of infectious peripheral vascular diseases, even though it has high rates of post-operational complications and mortality (Luo et al., 2003; Müller et al., 2001; Kyriakides et al., 2004). In a study by Muller et al. published in 2001 10, the mortality rate of open surgery for ruptured mycotic aneurysms of aorta and iliac arteries was as high as 63%. Similar to open surgeries for other infectious vascular diseases, the objective of open surgery for infected aneurysms caused by brucellosis is to resect infected lesions and necrotic tissues, and restore function. Compared with traditional open surgery, endovascular repair is a simpler, less invasive, and more effective procedure (Jiang et al., 2023; Jones et al., 2005; Ting et al., 2006; Clough et al., 2009). However, endovascular repair was once considered a contraindication for infected aortic and iliac aneurysms. The main concern was that the infection at the site of stent implantation could not be effectively cleared, which could easily lead to endograft infection, recurrence, and sepsis (Bakhos et al., 2010; Li et al., 2019; Sörelius et al., 2014). Endovascular repair of fungal aneurysms was first reported by Semba et al. (1998). Since endovascular repair was first used to treat infected aneurysm caused by brucellosis in 2007 (Kusztal et al., 2007), endovascular therapy has been increasingly used to treat this brucellosis complication, and it has proved to be highly efficacious. Although still under debate (Jiang et al., 2023), many believe that the use of covered stents in combination with antibiotics for *Brucella* infections is a viable treatment option for infected aneurysms caused by brucellosis. In the current review, 43.1% of patients (56/130) received endovascular surgery and 40.0% (52/130) received open surgery. Notably, the proportions of patients who received endovascular therapy within the past 5 and 2 years were 69.1% and 74.1%, respectively. This finding indicates that endovascular therapy has become a preferred option for surgical treatment over the years. In the current review, the mortality rate was 3.6% (2/56) for endovascular therapy, 15.4% (8/52) for open surgery, and 40.0% (6/15) for conservative treatment. These data support that endovascular intervention is a viable treatment option for aortic and iliac involvement in brucellosis.

The current review had a few limitations. First, this review only included cases that had already been published. As such, the results may be influenced by publication bias. Second, some articles included in this review did not provide data on the duration of symptoms, complications, duration of follow-up, or patient outcomes, which caused some degree of data uncertainty, especially in terms of mortality and survival. Third, the results may be limited by the small sample size. Prospective studies with a larger sample size are required to verify the efficacy and safety of endovascular techniques for the treatment of aortic and iliac involvement in brucellosis, even though this could be very difficult, given the extreme rarity of the condition.

## Conclusion

Aortic and iliac involvement in brucellosis is extremely rare but can be life-threatening. Its occurrence appears to be associated with the male gender, an older age, smoking history, and arteriosclerosis. Although the number of reported cases in developing countries has increased significantly in recent years, its incidence in these countries may still be underestimated. If a patient with an epidemiological history has persistent fever, chest pain, or abdominal pain that does not respond to conventional treatment, aortic and iliac involvement in brucellosis should be considered. Early diagnosis and therapeutic intervention with antibiotics and surgery are critical in improving patient outcomes. Endovascular therapy has become a preferred surgical treatment in recent years, and yet, its long-term complications remain to be assessed.

## Data Availability

The original contributions presented in the study are included in the article/Supplementary Material; further inquiries can be directed to the corresponding author.
